# Clinical Spectrum and Outcomes of Acute Upper Gastrointestinal Bleeding: A Prospective Comparative Study Between Elderly and Younger Adults

**DOI:** 10.7759/cureus.103849

**Published:** 2026-02-18

**Authors:** Mounika Kotte, Saad Manzoor, Hinza Hassan, Bismillah Athar Dar, Shanzey N Khan, Zeeshan Hameed, Muhammad Subhan, Gopi Sairam Reddy Mulaka, Seth Omari Mensah

**Affiliations:** 1 Internal Medicine, Knapp Medical Center, Weslaco, USA; 2 Internal Medicine, Islam Teaching Hospital, Sialkot, PAK; 3 Radiology, Ghurki Trust Teaching Hospital, Lahore, PAK; 4 Medical School, Lahore Medical and Dental College, Lahore, PAK; 5 Internal Medicine, Allama Iqbal Medical College, Lahore, PAK; 6 General Practice, Quaid-E-Azam Medical College, Bahawalpur, PAK; 7 General Practice, Rahbar Medical and Dental College, Lahore, PAK; 8 Internal Medicine, Jinnah Hospital Lahore, Lahore, PAK; 9 Internal Medicine, St. Martinus University Faculty of Medicine, Willemstad, CUW; 10 Obstetrics and Gynaecology, Royal Free Hospital, London, GBR

**Keywords:** elderly, endoscopy, mortality, non-elderly patient, rebleeding, rockall score, upper gastrointestinal (ugi) bleeding, variceal bleeding

## Abstract

Background

Upper gastrointestinal bleeding (UGIB) is a common medical emergency with age-related variation in etiology and outcomes. This study prospectively compared the clinical profile, endoscopic findings, and outcomes of UGIB in elderly (>60 years) and non-elderly (≤60 years) patients.

Methodology

A prospective observational study was conducted at a tertiary care hospital in Pakistan between December 1, 2023, and December 31, 2025. A total of 162 patients undergoing upper gastrointestinal endoscopy were equally stratified by age. Risk stratification was performed using the Rockall score, and outcomes, including rebleeding, transfusion requirements, and in-hospital mortality, were analyzed.

Results

Elderly patients had significantly higher Rockall scores (5.87 ± 1.89 vs. 3.68 ± 2.42; p < 0.001) and predominantly non-variceal bleeding, while non-elderly patients more frequently had variceal bleeding. Rebleeding occurred in one-third of patients, predominantly from variceal sources. Overall mortality was low (4.3%) and was associated with higher Rockall scores.

Conclusions

UGIB demonstrates distinct age-related patterns in etiology and severity. Rockall-based risk stratification and timely endoscopy remain essential tools for effective, age-tailored management.

## Introduction

Upper gastrointestinal bleeding (UGIB) is still one of the most frequent causes of medical emergencies in hospitals worldwide, with a high rate of morbidity and mortality and a substantial burden on the healthcare system [1]. Defined as bleeding from the gastrointestinal tract above the ligament of Treitz, UGIB typically manifests as vomiting blood, black tarry stools, or, in grave situations, fresh bleeding per rectum [2]. Notwithstanding the progress in the field of endoscopic hemostasis and the use of drugs, the death rate from this condition is still between 2% and 10%, which justifies the demand for efficient risk assessment and prompt treatment [3].

The etiology of UGIB varies considerably with age [4]. Younger patients frequently exhibit bleeding related to peptic ulcer disease or variceal hemorrhage, often associated with lifestyle factors such as alcohol consumption or nonsteroidal anti-inflammatory drug (NSAID) use [5]. In contrast, elderly patients (typically defined as >60 years) present distinct challenges due to age-related physiological changes, including reduced mucosal repair capacity, altered pain perception, and a higher prevalence of comorbidities [6]. Furthermore, polypharmacy, particularly the use of antiplatelet agents, anticoagulants, and NSAIDs, significantly elevates bleeding risk in this population [6]. These factors contribute to delayed diagnosis, more severe presentations, and poorer outcomes in the elderly compared to their younger counterparts [7]. Risk assessment is fundamental in UGIB management. The Rockall score, a tool that combines clinical and endoscopic factors, is a good predictor of rebleeding and death [8]. Similarly, the Child-Pugh-Turcotte (CPT) score provides essential prognostic data for patients with suspected variceal bleeding and liver disease [9]. Early endoscopy (<24 hours) is recommended for both diagnosis and therapeutic intervention, with modalities such as endoscopic variceal ligation, clipping, and thermal coagulation forming the mainstay of treatment [10].

While several studies from Western populations have delineated age-related differences in UGIB, data from the Indian subcontinent remain relatively limited [11,12]. Existing Asian studies have primarily focused on general UGIB epidemiology without a comprehensive age-stratified analysis [13,14]. Given the distinct sociodemographic, etiological, and clinical profiles in this region, including a high prevalence of portal hypertension and varied access to healthcare, there is a compelling need for focused research comparing UGIB in elderly versus non-elderly Asian patients [15]. The primary objective of this study was to compare the etiological spectrum and disease severity (Rockall score) of UGIB between elderly and non-elderly patients. Secondary objectives included comparison of clinical presentation, endoscopic interventions, rebleeding rates, transfusion requirements, length of hospital stay, and in-hospital mortality.

## Materials and methods

Study design and setting

This was a hospital-based, prospective observational study conducted to compare the clinical characteristics, etiological spectrum, and outcomes of UGIB between elderly (>60 years) and non-elderly (≤60 years) patients. The study was conducted in Pakistan over a 20-month enrollment period, from December 1, 2023, to December 31, 2025, at a tertiary care teaching hospital. Upper gastrointestinal endoscopy was performed according to standard institutional protocols by experienced gastroenterologists.

Study population

Inclusion Criteria

Adult patients (≥18 years) admitted with features suggestive of acute UGIB, including hematemesis, melena, or hematochezia, who underwent upper gastrointestinal endoscopy during hospitalization were included.

Exclusion Criteria

Patients were excluded if they had lower gastrointestinal bleeding confirmed by colonoscopy, bleeding from non-gastrointestinal sources (e.g., nasopharyngeal or pulmonary), known bleeding disorders unrelated to UGIB, contraindications to or non-performance of endoscopy, or refusal or inability to provide informed consent.

Sample size and group allocation

A total of 162 eligible patients were enrolled during the study period. Based on age at presentation, patients were stratified into the following two equal groups: elderly (>60 years; n = 81) and non-elderly (≤60 years; n = 81). As this was a prospective observational study, no a priori sample size calculation was performed; all eligible patients presenting during the study period were enrolled.

Data collection

Data were collected prospectively using a standardized case record form. Patients were enrolled consecutively to minimize selection bias. Cases with incomplete essential clinical or endoscopic data were excluded, and no imputation was performed for missing variables.

Patient characteristics

Demographic variables, presenting symptoms (hematemesis, melena, hematochezia), admission vital signs, comorbidities (hypertension, diabetes mellitus, chronic liver disease, chronic kidney disease), and medication history (antiplatelet agents, anticoagulants, NSAIDs) were recorded.

Clinical management

All patients received standard emergency management for UGIB, including intravenous fluid resuscitation, proton pump inhibitor therapy, and blood transfusions when indicated (hemoglobin <7 g/dL, or <8 g/dL in patients with cardiovascular disease). The timing of endoscopy was documented and categorized as early (≤24 hours) or delayed (>24 hours) after admission.

Laboratory investigations

Baseline laboratory parameters included complete blood count, coagulation profile (prothrombin time, activated partial thromboplastin time, international normalized ratio), liver function tests, and renal function tests, performed according to standard hospital laboratory protocols.

Endoscopic findings

Endoscopic variables included the primary source of bleeding, presence and grade of esophageal or gastric varices (grades 1-3), non-variceal lesions (peptic ulcers, erosions, malignancies), stigmata of recent hemorrhage, and endoscopic therapeutic interventions performed.

Risk stratification

The Rockall score was calculated for all patients using clinical and endoscopic parameters. The CPT score was calculated for patients with variceal bleeding to assess the severity of underlying liver disease. Both scoring systems are widely used, openly accessible clinical tools published in the scientific literature and do not require licensing permissions.

Outcome measures

Primary Outcomes

Comparison of UGIB etiology and Rockall scores between elderly and non-elderly patients.

Secondary Outcomes

Rebleeding (defined as recurrence of bleeding symptoms with a ≥2 g/dL drop in hemoglobin or requirement for repeat endoscopy), blood transfusion requirements, type of endoscopic intervention, length of hospital stay, and in-hospital mortality.

Statistical analysis

Statistical analysis was performed using SPSS version 26.0 (IBM Corp., Armonk, NY, USA). Continuous variables were expressed as mean ± standard deviation and compared using Student’s t-test. Categorical variables were expressed as frequencies and percentages and compared using the chi-square or Fisher’s exact test. A p-value <0.05 was considered statistically significant. Multivariable logistic regression analysis was performed using clinically relevant variables, including age group, Rockall score, bleeding etiology, and timing of endoscopy. Given the limited number of mortality events, regression results, particularly for mortality, were interpreted cautiously.

## Results

**Figure 1:**
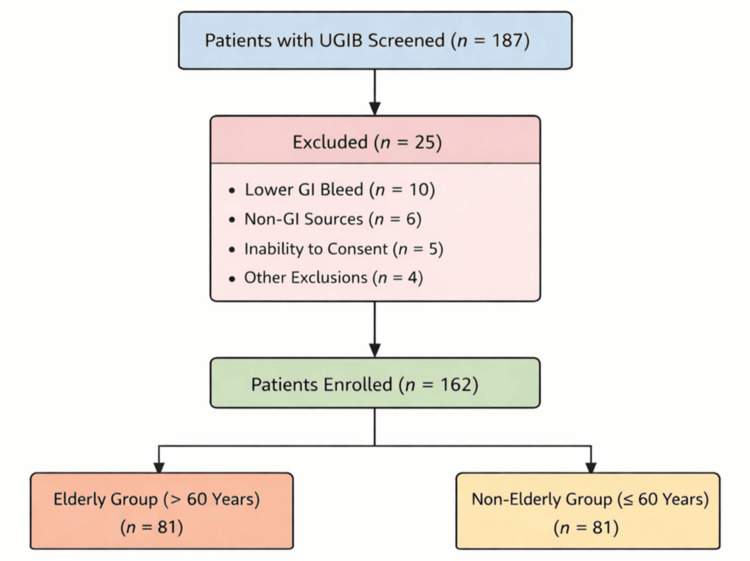
Flow diagram illustrating patient selection. UGIB: upper gastrointestinal bleeding; GI: gastrointestinal

During the 20-month study period, 187 patients presenting with symptoms of UGIB were screened, of whom 162 met the inclusion criteria and were enrolled (Figure 1).

Patients were stratified equally into elderly (>60 years; n = 81) and non-elderly (≤60 years; n = 81) groups. Baseline demographic and clinical characteristics are summarized in Table 1.

**Table 1:** Baseline demographic and clinical characteristics. *: Defined as systolic BP <100 mmHg or heart rate >100 beats/minute. NSAIDs: non-steroidal anti-inflammatory drugs; BP: blood pressure

Category	Characteristic	Elderly (n = 81)	Non-elderly (n = 81)	Test/Mean value	P-value
Demographics	Age, years (mean ± SD)	68.4 ± 6.2	42.3 ± 11.8	t = 18.45	<0.001
	Male gender, n (%)	60 (74.1%)	64 (79.0%)	χ² = 0.58	0.447
Comorbidities	Hypertension, n (%)	48 (59.3%)	22 (27.2%)	χ² = 17.60	<0.001
	Diabetes mellitus, n (%)	36 (44.4%)	18 (22.2%)	χ² = 9.46	0.002
	Coronary artery disease, n (%)	22 (27.2%)	8 (9.9%)	χ² = 8.38	0.004
	Chronic liver disease, n (%)	29 (35.8%)	41 (50.6%)	χ² = 3.62	0.058
	Chronic kidney disease, n (%)	14 (17.3%)	6 (7.4%)	χ² = 3.81	0.051
Medication use	Antiplatelets, n (%)	32 (39.5%)	12 (14.8%)	χ² = 13.13	<0.001
	Anticoagulants, n (%)	18 (22.2%)	6 (7.4%)	χ² = 6.99	0.008
	NSAIDs, n (%)	24 (29.6%)	28 (34.6%)	χ² = 0.46	0.498
Clinical presentation	Hematemesis, n (%)	35 (43.2%)	52 (64.2%)	χ² = 7.42	0.007
	Melena, n (%)	48 (59.3%)	36 (44.4%)	χ² = 3.90	0.048
	Hematochezia, n (%)	8 (9.9%)	12 (14.8%)	χ² = 0.90	0.342
	Hemodynamic instability, n (%)	22 (27.2%)	18 (22.2%)	χ² = 0.53	0.468

The mean age was significantly higher in the elderly group (68.4 ± 6.2 years) compared to the non-elderly group (42.3 ± 11.8 years; t = 18.45, p < 0.001). Male predominance was observed in both groups without a significant difference (elderly: n = 60, 74.1% vs. non-elderly: n = 64, 79.0%; χ² = 0.58, p = 0.447). Elderly patients had a significantly higher prevalence of comorbidities, including hypertension (n = 48, 59.3% vs. n = 22, 27.2%; χ² = 17.60, p < 0.001), diabetes mellitus (n = 36, 44.4% vs. n = 18, 22.2%; χ² = 9.46, p = 0.002), and coronary artery disease (n = 22, 27.2% vs. n = 8, 9.9%; χ² = 8.38, p = 0.004). Chronic liver disease was more frequent in non-elderly patients, although this difference did not reach statistical significance (n = 41, 50.6% vs. n = 29, 35.8%; χ² = 3.62, p = 0.058). Use of antiplatelet agents (n = 32, 39.5% vs. n = 12, 14.8%; χ² = 13.13, p < 0.001) and anticoagulants (n = 18, 22.2% vs. n = 6, 7.4%; χ² = 6.99, p = 0.008) was significantly higher in elderly patients. NSAID use was comparable between groups (n = 24, 29.6% vs. n = 28, 34.6%; χ² = 0.46, p = 0.498). Regarding clinical presentation, hematemesis was more common in non-elderly patients (n = 52, 64.2% vs. n = 35, 43.2%; χ² = 7.42, p = 0.007), whereas melena was more frequent in elderly patients (n = 48, 59.3% vs. n = 36, 44.4%; χ² = 3.90, p = 0.048). Hemodynamic instability at presentation did not differ significantly between groups (n = 22, 27.2% vs. n = 18, 22.2%; χ² = 0.53, p = 0.468).

Risk stratification scores

The mean Rockall score was significantly higher among elderly patients compared to non-elderly patients (5.87 ± 1.89 vs. 3.68 ± 2.42; t = 6.32, p < 0.001). When stratified by bleeding etiology, elderly patients with variceal bleeding demonstrated the highest Rockall scores (7.48 ± 0.87). CPT scores did not differ significantly between elderly and non-elderly patients with non-variceal bleeding (5.81 ± 1.55 vs. 5.75 ± 1.37; t = 0.19, p = 0.853). Among patients with variceal bleeding, elderly patients had slightly lower CPT scores compared to non-elderly patients, though this difference was not statistically significant (7.86 ± 2.56 vs. 8.93 ± 2.41; t = 1.77, p = 0.081).

Laboratory parameters

Laboratory parameters stratified by bleeding etiology are presented in Table 2.

**Table 2:** Selected laboratory parameters by bleeding etiology. Values are expressed as mean ± standard deviation. *: Statistically significant (p < 0.05). AST: aspartate aminotransferase

Parameter	Elderly (non-variceal, n = 52)	Non-elderly (non-variceal, n = 40)	t-value	P-value	Elderly (variceal, n = 29)	Non-elderly (variceal, n = 41)	t-value	P-value
Hemoglobin day 1 (g/dL)	8.76 ± 3.53	9.31 ± 3.43	−0.75	0.456	8.92 ± 1.75	8.74 ± 2.56	0.33	0.740
Platelets (×10³/μL)	237.6 ± 81.3	243.1 ± 116.2	−0.27	0.791	130.8 ± 61.8	116.8 ± 98.1	0.68	0.498
Blood urea (mg/dL)	64.17 ± 47.76	44.79 ± 46.13	1.92	0.057	48.07 ± 22.74	38.80 ± 32.67	1.30	0.199
AST (U/L)	41.63 ± 56.77	46.54 ± 47.65	−0.43	0.668	51.31 ± 34.69	105.24 ± 101.22	−3.15	0.008*

Among patients with non-variceal bleeding, elderly patients had higher blood urea levels compared to non-elderly patients, approaching statistical significance (64.17 ± 47.76 vs. 44.79 ± 46.13 mg/dL; t = 1.92, p = 0.057). In patients with variceal bleeding, aspartate aminotransferase levels were significantly lower in elderly patients compared to non-elderly patients (51.31 ± 34.69 vs. 105.24 ± 101.22 U/L; t = -3.15, p = 0.008). No significant differences were observed between groups for hemoglobin levels, platelet counts, renal function, or coagulation parameters.

Endoscopic findings

Endoscopic diagnoses are summarized in Table 3.

**Table 3:** Endoscopic diagnoses. *: Statistically significant (p < 0.05).

Diagnosis	Elderly (n = 81)	Non-elderly (n = 81)	Test statistic	P-value
Variceal causes, n (%)	32 (39.5%)	43 (53.1%)	χ² = 3.11	0.078
Grade 3 esophageal varices, n (%)	24 (29.6%)	29 (35.8%)	χ² = 0.72	0.398
Non-variceal causes, n (%)	40 (49.4%)	24 (29.6%)	χ² = 7.37	0.007*
Gastric erosions/ulcer, n (%)	18 (22.2%)	7 (8.6%)	χ² = 5.73	0.017*
Duodenal ulcer, n (%)	10 (12.3%)	8 (9.9%)	χ² = 0.26	0.611
Miscellaneous, n (%)	9 (11.1%)	14 (17.3%)	χ² = 1.28	0.256

Variceal bleeding was more common among non-elderly patients (n = 43, 53.1% vs. n = 32, 39.5%), whereas non-variceal bleeding predominated in elderly patients (n = 40, 49.4% vs. n = 24, 29.6%; χ² = 7.37, p = 0.007). Grade 3 esophageal varices were the most frequently observed variceal lesion in both groups. Among non-variceal etiologies, gastric erosions or ulcers were significantly more common in elderly patients (n = 18, 22.2% vs. n = 7, 8.6%; χ² = 5.73, p = 0.017). Other non-variceal causes, including duodenal ulcers and miscellaneous lesions, were similarly distributed between groups.

Endoscopic interventions

Endoscopic variceal ligation was the most frequently performed intervention overall (n = 54, 33.3%), with a higher utilization in non-elderly patients, although the difference was not statistically significant (n = 32, 39.5% vs. n = 22, 27.2%; χ² = 2.97, p = 0.085). Hemoclipping was used significantly more often in elderly patients (n = 7, 8.6% vs. n = 1, 1.2%; χ² = 4.59, p = 0.032). Other therapeutic modalities, including sclerotherapy, argon plasma coagulation, and cyanoacrylate glue injection, were used with comparable frequency between groups.

Clinical outcomes

**Figure 2:**
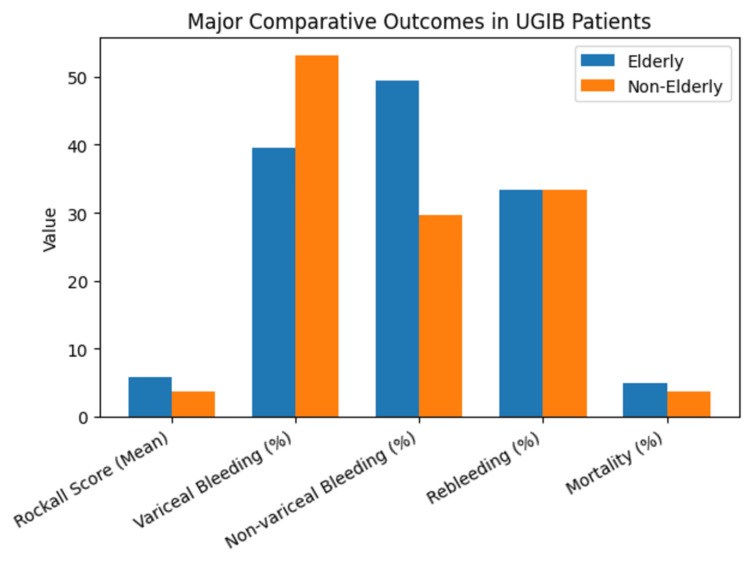
Summary of major comparative outcomes in elderly and non-elderly patients with upper gastrointestinal bleeding. UGIB: upper gastrointestinal bleeding

Rebleeding occurred in 54 (33.3%) patients, with no significant difference in overall incidence between elderly and non-elderly groups. However, rebleeding due to variceal sources was more frequent in non-elderly patients (n = 20, 74.1% of their rebleeds vs. n = 15, 55.6% of rebleeds in the elderly; χ² = 4.18, p = 0.041). Packed red blood cell transfusion requirements were similar between groups (elderly: n = 40, 49.4% vs. non-elderly: n = 36, 44.4%; χ² = 0.40, p = 0.526). In-hospital mortality occurred in seven (4.3%) patients, including four elderly (4.9%) and three non-elderly (3.7%) patients, with no significant difference between groups (χ² = 0.15, p = 0.700). All deaths were attributed to hypovolemic shock secondary to massive UGIB. Patients who died had significantly higher Rockall scores compared to survivors (7.86 ± 1.07 vs. 4.92 ± 2.31; t = 4.95, p < 0.001). Figure 2 provides a graphical representation of these major outcomes.

Timing of endoscopy

Early endoscopy (≤24 hours) was performed in 41 (25.3%) patients, with similar rates in elderly and non-elderly groups (n = 21, 26.3% vs. n = 20, 24.2%; χ² = 0.11, p = 0.742). Patients undergoing early endoscopy demonstrated lower rebleeding rates (10 of 41 patients, 24.4% vs. 44 of 121 patients, 36.4%; χ² = 1.97, p = 0.160) and significantly shorter hospital stays (5.2 ± 2.1 vs. 6.8 ± 3.4 days; t = -2.84, p = 0.005).

Multivariate analysis

On multivariate logistic regression analysis, independent predictors of rebleeding included a Rockall score ≥6 (odds ratio (OR) = 3.42; 95% confidence interval (CI) 1.78-6.58; p < 0.001), variceal bleeding etiology (OR = 2.16; 95% CI = 1.12-4.17; p = 0.022), and delayed endoscopy (>24 hours) (OR = 1.89; 95% CI = 1.02-3.51; p = 0.044). For in-hospital mortality, only a Rockall score ≥7 independently predicted death (OR = 5.24; 95% CI = 2.31-11.89; p < 0.001).

## Discussion

This study identified disparate clinical phenotypes of acute UGIB in a Pakistani tertiary care setting, revealing significant age-related differences in etiology, severity, and presentation. The older group of patients (>60 years) mostly experienced non-variceal bleeding (n = 40, 49.4%), which was at least partly explained by the increased use of antiplatelet agents in this group, and had substantially higher Rockall scores (5.87 vs. 3.68, p = 0.0005), indicating greater comorbidity. On the other hand, younger patients (≤60 years) were more likely to have variceal bleeding (n = 43, 53.1%), thus mirroring the local epidemiology of chronic liver disease [1]. These results are in good agreement with patterns observed in other South Asian populations and clearly indicate the need for age-specific diagnostic and therapeutic strategies [1,16].

Directly from the etiological differences stemmed the different management tactics. Endoscopic variceal ligation, the first-line therapy for variceal bleeding according to guidelines, was the most frequently performed procedure (n = 54, 33.3%) [6]. The substantially higher usage of hemoclipping in the elderly group (n = 7, 8.6%) mainly targeted their increased proportion of ulcer-related bleeding; thus, this practice was in line with standard endoscopic treatments for non-variceal lesions [13]. One of the major findings was the low proportion of endoscopy within 24 hours of admission (n = 41, 25.3%). Although this is below the guideline recommendations, the association found between early endoscopy and shorter hospital stay points to a very important target for quality improvement. Recently, evidence has shown that a pragmatic focus on endoscopy within 24 hours is essential, as “urgent” endoscopy (<6 hours) may not see a mortality advantage over “early” endoscopy (6-24 hours) for all cases, thus supporting a feasible standard setting in resource-limited environments [11].

However, risk stratification remains a major issue. The Rockall score identified high-risk elderly patients in our cohort, a utility that was externally validated and strongly correlates with rebleeding and mortality [17]. The Rockall score is the most popular in clinical practice. However, recent studies demonstrate the usefulness of other instruments, such as the Glasgow and Blatchford scores, which are used for pre-endoscopic triage, and of new prognostic markers, such as the blood urea nitrogen-to-creatinine ratio [17,18]. The high overall rebleeding rate noted in our study (n = 54, 33.3%), especially from variceal sources, is consistent with the fact that portal hypertension remains a therapeutic challenge [6]. Furthermore, it might still be the case that the elderly group with higher Rockall scores did not have significantly higher hospital mortality than the younger group because mortality in this elderly population is of a different nature. It has been shown that death in elderly UGIB patients is mostly caused by decompensation of underlying comorbidities (e.g., sepsis, cardiac events) rather than exsanguination; the focus of treatment is not only achieving hemostasis but also managing the patient’s comorbidities [7]. The high and almost equal need for blood transfusion in the two groups (elderly: n = 40, 49.4%; non-elderly: n = 36, 44.4%) highlights the considerable resource demand caused by UGIB. It also serves as a reminder of the necessity of strictly following evidence-based, restrictive transfusion protocols [14].

In short, this research verifies that UGIB presents two main age-related syndromes in our region. Treatment should thus be differentiated: prompt consideration of variceal causes in the young and a thorough evaluation of the elderly patient’s medications/health conditions. The Rockall score is an effective tool for risk prediction; however, combining it with other scores still needs to be tested in our local setting [15,17-20]. Key factors for improving prognosis include prioritizing endoscopy within 24 hours and actively treating comorbidities.

Limitations of this single-center study include its modest sample size, particularly the small number of mortality events (n = 7, 4.3%), which limits the robustness of mortality comparisons and modeling, as well as its observational design regarding the timing of endoscopy. The single-center nature of the study restricts generalizability, and the observational design precludes causal inference. Additionally, residual confounding from comorbidities, medication use, and timing of endoscopy cannot be excluded. Prospective multicenter studies are required to confirm age-specific management pathways and to optimize resource utilization in our region.

## Conclusions

This research reveals that there are clearly different UGIB profiles between elderly and non-elderly patients in a tertiary care setting. Elderly patients are characterized by higher Rockall scores and more non-variceal bleeding (mostly linked to the use of antiplatelet/anticoagulants), and they also have different symptom patterns. Variceal bleeding makes up the majority of cases in non-elderly patients, who usually present with more dramatic hematemesis. The Rockall score is a useful tool for predicting the likelihood of rebleeding and death in both groups. Despite these differences, the ways patients were treated and their short-term results did not differ greatly. However, there was still room for improvement in patient care through early endoscopy and age-tailored diagnostic considerations. These results emphasize the need for age-specific methods for UGIB and underscore the importance of validated risk scores in decision-making.
